# The impact of cross-kingdom molecular forensics on genetic privacy

**DOI:** 10.1186/s40168-021-01076-z

**Published:** 2021-05-20

**Authors:** Eran Elhaik, Sofia Ahsanuddin, Jake M. Robinson, Emily M. Foster, Christopher E. Mason

**Affiliations:** 1 Department of Biology, Lund University, 22362 Lund, Sweden; 2 Department of Medical Education, Icahn School of Medicine at Mount Sinai, New York, USA; 3 The Department of Landscape Architecture, University of Sheffield, Sheffield, S10 2TN UK; 4 The Healthy Urban Microbiome Initiative (HUMI), Adelaide, 5005 South Australia; 5 Columbia, USA; 6 Department of Physiology and Biophysics, Weill Cornell Medicine, New York, NY 10021 USA; 7 The HRH Prince Alwaleed Bin Talal Bin Abdulaziz Alsaud Institute for Computational Biomedicine, New York, NY 10021 USA; 8 The Feil Family Brain and Mind Research Institute (BMRI), New York, NY 10021 USA; 9 The Information Society Project, Yale Law School, New Haven, CT 06511 USA

**Keywords:** Genomics, Genetic privacy, Metagenomics, Next-generation sequencing, Forensics, Genetic discrimination, Microbiomics

## Abstract

Recent advances in metagenomic technology and computational prediction may inadvertently weaken an individual’s reasonable expectation of privacy. Through cross-kingdom genetic and metagenomic forensics, we can already predict at least a dozen human phenotypes with varying degrees of accuracy. There is also growing potential to detect a “molecular echo” of an individual’s microbiome from cells deposited on public surfaces. At present, host genetic data from somatic or germ cells provide more reliable information than microbiome samples. However, the emerging ability to infer personal details from different microscopic biological materials left behind on surfaces requires in-depth ethical and legal scrutiny. There is potential to identify and track individuals, along with new, surreptitious means of genetic discrimination. This commentary underscores the need to update legal and policy frameworks for genetic privacy with additional considerations for the information that could be acquired from microbiome-derived data. The article also aims to stimulate ubiquitous discourse to ensure the protection of genetic rights and liberties in the post-genomic era.

Video abstract

**Supplementary Information:**

The online version contains supplementary material available at 10.1186/s40168-021-01076-z.

## Background

DNA sampling and sequencing are now routinely applied in several areas of research and practice. These include criminal investigations, research on public parks and subways, and multidisciplinary genomics projects. The collection of genomic and metagenomic profiles across the world has led to a greater understanding of the world’s genetic diversity. However, it also raises an array of ethical and legal questions [1–4]. Indeed, privacy expectations are reduced once personal materials are in the public realm. A person’s genetic information (e.g., acquired from their hair, skin cells, or microbiome) could conceivably be collected in a public setting. This could have a considerable impact on privacy, and the ability to genetically discriminate could be utilized for nefarious means.

In the USA, the Genetic Information Non-discrimination Act 2008 (GINA) made it illegal for companies with over 15 employees to use genetic discrimination for health insurance and employment purposes [5]. However, a person and their family’s genetic profiles can still be utilized to deny or adjust life insurance, long-term care insurance, and disability insurance [6]. Survey data show that 6 years after its enactment, over 80% of US adults were unaware of GINA, and 30% of those who were informed reported deep concerns about genetic discrimination [5]. The National Institute of Health’s “Precision Medicine Initiative” [7] and genome-guided medical care also raise concerns regarding privacy and safety. This is due to the risks of data sharing when congenital haplotypes found in the data may be used to discriminate against patients and their family members. In addition, the depth of information gained from genetic remnants on public surfaces has implications for individual rights to genetic privacy under GINA and the criminal justice system.

In 2019, an investigation was launched into the supply of 1500 DNA samples from the Crumlin children’s hospital in Ireland to a DNA collection company without authorization from the patients [8]. This may represent a breach of the European Union’s general data protection regulation (GDPR) Article 9, which requires proper consent for the processing of DNA data [9]. However, many countries implement their own interpretation of this regulation, thus leading to calls to develop a cross-border code of conduct for genomic data sharing [10]. Importantly, explicit considerations for microbiome-derived information are not part of the genetic privacy narrative that informs this regulation. In March 2018, a Parliamentary Joint Committee (PJC) inquiry into the Australian life insurance industry recommended an immediate ban on the use of predictive genetic test results [11]. A recent study revealed illegal genetic discrimination by Australian life insurance companies, representing a broader concern [11].

In criminal law, the US legal system’s notion of privacy under the Fourth Amendment is based on a person’s reasonable expectation of privacy: “what a person knowingly exposes to the public, even in his own home or office, is not a subject of Fourth Amendment protection [12].” The prevailing question is should humans, who constantly shed their genetic (host and microbiome) materials, have a reasonable expectation that these materials will not be collected from public surfaces? In other words, should genetic material be treated differently than fingerprints or material property? As we enter the era of “ubiquitous sequencing [2],” people must now assume that their DNA can be collected, sequenced, annotated, and interpreted by other people, particularly if retrievable from a public place. In that respect, another important question is when a person leaves DNA or RNA behind on a public surface, what personal information could be revealed?

## Main text

### Molecular signatures in the post-genomic era

**Fig. 1:**
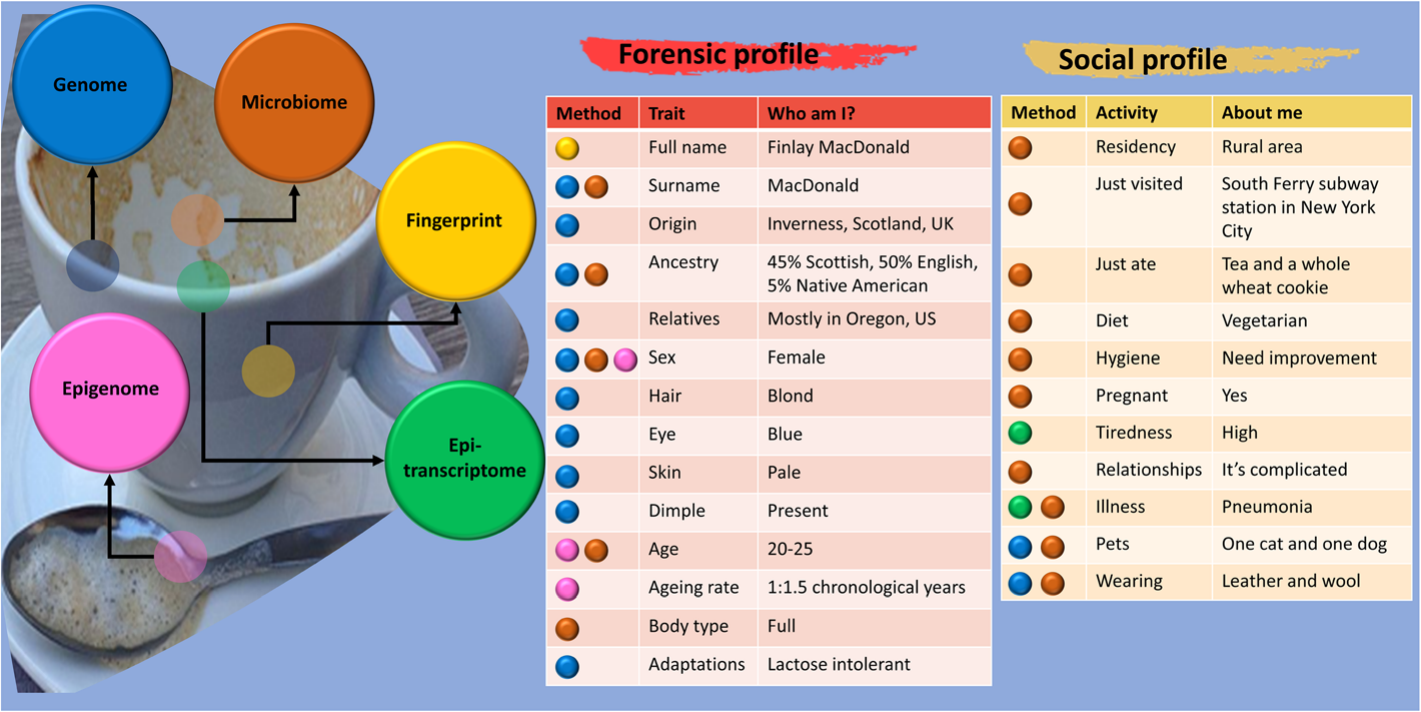
Cross-kingdom methods of forensics. Categories of multi-omic and multi-kingdom measurements can create both forensic (left table) and social (right table) profiles for a person, based on their epigenome (pink), epitranscriptome (green), fingerprint (yellow), microbiome (orange), and genome (blue). Categories for types of inferred information are detailed in each table by the trait/activity and the revelatory information

Technological advances in genomics have created the potential for forensic methods beyond DNA, leading us to a “post-genomic” forensic era with identifiable information in the RNA and epigenetic states. Information that can contribute to identifying human individuals could potentially be found within the vast diversity of microorganisms that are in, on, and around us, collectively known as the microbiome [13, 14]. Next-generation sequencing (NGS) has been used to localize these organisms to a unique part of the human body, forming a “molecular cartography” of an individual [15]. Beyond microorganisms, recent metagenomics studies have enabled a cross-kingdom examination of life, including urban genetic maps [16] in worldwide cities [17]. The availability of these new datasets and methods can enable inferences of at least a dozen phenotypes across several categories. These include human DNA, human RNA, epigenetics, epitranscriptome, and metagenomes (Fig. 1). Furthermore, the advent of “Big Data” allows the intersection of these identifiers to provide additional revelatory information.

In the following 12 sections, we summarize the phenotypes that could be discerned from DNA or RNA deposited on public surfaces, along with methods to acquire this information. Some of the suggestions are still in development, but given the ubiquity of genomic tools and possibilities for advancements, it is still important to highlight areas with potential.

#### Identity

Criminal cases have been solved by comparing DNA found at the crime scene with the DNA of individuals or their families who have submitted their genomic data to genealogy databases [18]. However, a supposedly “anonymous dataset” may also reveal a person’s identity using the Y-chromosome’s short tandem repeats (STRs), which can be linked to surnames [19, 20], or by querying genealogy databases [21], which arguably is constitutional under the Fourth Amendment [22]. The widespread use of DNA profiling with Combined DNA Index System (CODIS) markers for anyone who is arrested allows law enforcement to use STR markers to find a relative who committed a crime through “familial DNA searches” [23]. Assuming a database of gut metagenomes from individuals, variation in gut microbiota can contribute towards genomic fingerprinting [24], which can be crossed with consumer DNA that companies like FamilyTreeDNA are sharing with the FBI [25]. Studies have shown that the skin microbiome may exhibit a degree of inter-individual variability and can be recovered from objects such as computers even if left untouched for 2 weeks [26]. The microbiome has also been linked to a person’s phone and shoes [27]. Many of the studies in the microbial forensics realm have moderate model accuracies, and improvements are required before highly accurate information can be elucidated. However, as technology continues to improve, there is potential to identify individuals through microbial profiles with a much higher accuracy level in the near future.

#### Age

Each time a cell replicates, a chromosomal loss can occur. Measuring these molecular alterations in the Y- and X-chromosomes [28], telomeres [29], and DNA methylation [29] allows the molecular age of the body to be estimated. Applications of the latter method have been reported to predict chronological age [29] closely. The skin microbiome also has the potential to be a predictor of age with high accuracy (mean ± standard deviation, 3.8 ± 0.45 years of chronological age) [30]; however, the results vary by tissue, gender, and age. Further studies are needed to demonstrate the applicability of this approach.

#### Biological sex

This can be inferred through DNA, RNA, and epigenetics [31]. The microbial communities of pubic hairs [32] and the lower gastrointestinal tract [33] also differ between the sexes. Moreover, Luongo et al. [34] studied airborne microbial diversity in university dormitory rooms (*n* = 91). Through relative abundance analysis, machine learning techniques could predict the biological sex of occupants with 79% accuracy [34]. As microbial relative abundance data are a completely different form of data to that of host DNA, it could contribute towards gaining personal information in the absence of quality host DNA samples.

#### Facial features

Several facial features, like hair color and texture/thickness, eye color, and skin tone, can be genetically predicted with varying levels of accuracy [23], and this is expected to improve as genotypic and phenotypic databases continue to increase.

#### Ancestry and biogeography

Differences between continental groups are observable in different phenotypes, such as genomic sequences [35] and the vaginal microbiome [36]. Genetic variation between human populations is engraved in ancestry informative markers (AIMs) [37], which can be used to predict the geographic region of the DNA’s ancestral origins with an accuracy of a few hundred kilometers [38] or less [39, 40]. However, caution should be practiced since biases may arise primarily due to the inaccuracy of ancestry estimation tools and subpopulation undersampling [41].

#### Geospatial localization

Various techniques are available to ascertain concurrent localization information, including microbial DNA of soil [42], microbial DNA of surfaces [14], pathogenic viruses [43], and metagenomes [44]. To geo-localize a sample, these approaches require global city-wide reference data, such as those collected by the MetaSUB consortium for metagenomes [14]. Although studies that aim to localize samples typically suffer from modest sample sizes or prioritize classification over prediction, there is undoubtedly potential to develop more rigorous methods and improve the confidence of geospatial inferences.

#### Zooculture

Metagenomes could provide valuable evidence regarding interactions with pets and wildlife. This information could conceivably be evidence of criminal behaviors such as illegal fishing or poaching [45]. Additionally, the exchange of microbial communities between a pet and the owner can provide information on the length of their association [46]. There are pathways to trace microbial profiles from other non-human species to a given environment or pet ownership. These ideas are theoretical at the moment, but with methodological refinement, there is potential to connect an individual with a particular location through their shared microorganisms with animals [47].

#### Cell type

The Human Microbiome Project (HMP) [13] and the assignment of bacteria to primary areas of the human body prompted the identification of body parts that may have been in contact with public surfaces in subways [16]. As each tissue also has unique gene expression [48], RNA [49], and epigenetic [50] measurements, different molecules could potentially be used for cross-validation in the future. This is currently unrealistic outside of lab conditions, but given the rapidity of technological advancements and model accuracies, the potential is considerable.

#### Obesity

Microbiome profiles of obese and non-obese people can differ dramatically [51].

This could not only allow the stratification of individual profiles [52], but also potentially provide insight into valuable information on digestion or the eating habits of the individual [53]. Although host genetics are more reliable for host identification, there is potential to acquire some useable information in the absence of sufficient quality and quantity of DNA from somatic or germ cells.

#### Pregnancy

The maternal gut microbiome begins similar to that of non-pregnant women [54] but changes throughout the pregnancy [55]. While the statistical relationships in these studies can be weak and multifactorial, they could, eventually, be used to identify an individual’s gestational status accurately.

#### Circadian rhythm

Although this application is presently only speculative, the coupling of RNA modifications with circadian rhythm could be developed as epitranscriptomic markers to determine if someone was sleeping or awake based on the cells left in their bed [56]. However, areas for advancement would need to include optimizing the processing of low biomass samples, which face additional challenges of degradation and environmental contamination.

#### Disease and infection

The carriage of oral pathogens is not only an indicator of periodontal health [57], but also an indicator of more complex disorders like pancreatic cancer [58]. Assuming RNA is preserved on a surface, modifications to RNA can contribute towards understanding if a person’s body is responding to a human immunodeficiency virus (HIV) infection, as well as the type and severity of the HIV infection [59]. It is unrealistic to assume that information can currently be used with a high level of accuracy. Still, it does highlight another potential privacy issue that could have important implications in the future.

### Ubiquitous measurements and potential counter-measures

Many recent advances in genomics and biomedicine are made possible by technological advancements in cameras and imaging. These tools have enabled us to peer inside single cells with unparalleled resolution. The affordability and high resolution of contemporary cameras have also enabled their adaptations to forensics. A “time machine” of movement around a city can be created with the use of drones that maintain a geosynchronous location above a city through daily image collection. A private company, Persistent Surveillance, has used manned aircraft equipped with high-powered cameras to track and catch criminals in Camden (New Jersey, USA) and Ciudad Juárez (Mexico) [60]. Location information can also be gained through cell phone tracking using the multilateration of radio signals between different cell towers of the network and the phone or even more easily using GPS. In 2019, the existence of a file containing 50 billion data points recording the movements of 12 million American cell phone users, including the US President and his guests, was revealed [61]. In a landmark US Supreme Court case, it was determined that “the time-stamped data provides an intimate window into a person’s life, revealing not only his particular movements but through them his ‘familial, political, professional, religious and sexual associations’” and is thereby a violation of the Fourth Amendment to the US Constitution to gain access to these data without a search warrant [62]. Personal information, however, continues to be collected, analyzed, and cross-referenced, as has been demonstrated when a teenager’s consumer metadata revealed her pregnancy even before her parents were made aware [63].

The new abilities to collect personal information have severe implications for people’s expectations of privacy. DNA evidence, the key to the conviction or exoneration of suspects of various types of crime, from theft to rape and murder, can be fabricated and planted in the crime scene to implicate any known genetic profile [64]. If travel history is detectable, it can not only be mined for criminal or other investigations but could potentially be used in marital disputes, employment discrimination, and other tracking purposes. Combined with data mining methods employed by corporations, surveillance tools are increasingly eliminating the traditional notion of privacy. Indeed, if DNA sequencers were truly ubiquitous, not only could one’s identity be matched with their location, but their actions and associations may also be inferred through a “genetic time machine” (Fig. 1) and cross-referenced with corporate and online information.

Due to these potential encroachments on privacy and ample molecular means to link deposited cells and molecules to phenotypes, new approaches for obfuscation have been developed and even patented [65]. It is possible to order and spray synthetic oligonucleotides to mask one’s historical presence in a room [64]. However, unless such “genetic camouflage” encompasses the cross-genome spectrum (Fig. 1), the deception could be detected. Nonetheless, the potential to assemble such a precise molecular match to a person’s genetic and molecular identity, which can be obtained from public databases [21], may create new opportunities in forensics and considerable problems. A technology for accurate and specific matches at the genetic, epigenetic, RNA, epitranscriptome, and microbial levels would also augment the ability to frame a person in a criminal context. Moreover, although this is currently speculative, with precise methods for trans-differentiation and re-differentiation of cells, it might be possible to convert the skin cells left behind at the scene of a crime into erythrocytes and imply that a bloody incident had occurred. With easy-to-use CRISPR systems [66] and epigenetic modification mechanisms [67], such precise and potentially malicious genetic manipulations can feasibly be developed.

Given these developments, we call for renewed and widespread discussions on genetic privacy, the legal implications of these new technologies, and updated statutory protections. We also call for explicit considerations for microbiome-derived data in any standards and regulations designed to protect genetic privacy.

## Conclusions

Just as the amount of traceable electronic data for people in the modern era only grows with time, the likelihood of a person’s biological remnants being found in the environment also increases with time, concomitant with an ever-increasing ability to extract and interpret these data. Many of the tools are theoretical at present, and molecular methods and interpretation are unlikely ever to be 100% accurate. Nonetheless, given the number of tests that can be used across all different data types, metrics, and kingdoms of life, a new “cross-kingdom forensic landscape” has emerged that eviscerates previous notions of privacy. The speed and availability of these tools, algorithms, and multi-layered mechanisms for detection and re-programming of identity from genomics and post-genomic data are increasing. This will likely create challenges for judicial enforcement for those who violate the law. It also raises critical questions about who has access to the metadata, genetic material, and the unintended revelatory information encased in microscopic particles left on every surface.

Given all the identifiable information that is present in a sample and all the metadata about people that are being collected, a new risk of discrimination is now an issue. Unfortunately, legal frameworks, like GINA [6] and equivalent US state statutes, do not prevent life insurance underwriters from changing their premiums based on genetic markers—even if the markers were taken from genetic material left on a drinking cup (Fig. 1) or the saliva under the stamp or envelope that was mailed to the insurance company [68]. Moreover, any information about family members could also *legally* be used as a basis for altered eligibility, coverage, or premiums on life, disability, or long-term care insurance. By extension, any of the forensics mechanisms described above could potentially be used to change, deny, or alter coverage for a person or their relatives.

These legal frameworks, designed to safeguard worker’s DNA and genetic information, are frozen by the definitions provided by the legislature and thus do not apply to the epigenome, microbiome, or metagenome information. Specifically, the GINA statute says that “a genetic test means an analysis of human DNA, RNA, chromosomes, proteins, or metabolites, that detects genotypes, mutations, or chromosomal changes” [5]. In other words, all other biologic non-genomic personal identifying information may be used to achieve what GINA attempted to prevent: health insurance and employment discrimination. Consider, for example, the case of *Lowe v. Atlas Logistics Group Retail Services* [69], where the company’s employees were asked to undertake a genetic Short tandem repeats (STR) test to identify the mysterious “devious defecator” who violated their warehouse. Plaintiffs Lowe and Dennis filed a lawsuit under GINA and won; however, had the plaintiffs been asked to submit a gut microbiome sample, GINA would not have been protected them. Concerningly, even the Patient Protection and Affordable Care Act (ACA) of 2010, which prohibits health insurance companies from using genetic information to establish the rules or terms of an individual’s eligibility, never defined “genetic information” [70].

It is noteworthy that the GINA and ACA only set the minimum bar of protection against genetic discrimination, and US state laws can set up stricter protections. For example, in 2011, the California Legislature passed the California Genetic Information Nondiscrimination Act” (CalGINA), yet even this more stringent act [71] maintained GINA’s definition for genetic information. Given this loophole, an updated GINA and similar frameworks should account for these non-human markers and the battery of molecular signatures described above. Only more inclusive policies that guard any “personally-identifying molecular signature” to be exempt from use in insurance and employment decisions and options for people, as well as their relatives, can guarantee against genetic discrimination. An example for such an elaborated definition can be found in the recently issued California Genetic Information Privacy Act (“GIPA”) aimed to regulate the privacy and security aspects of genetic testing and testing companies. The act defines “Genetic data” as “any data, regardless of its format, that results from the analysis of a biological sample from a consumer, or from another element enabling equivalent information to be obtained, and concerns genetic material. Genetic material includes, but is not limited to, deoxyribonucleic acids (DNA), ribonucleic acids (RNA), genes, chromosomes, alleles, genomes, alterations or modifications to DNA or RNA, single nucleotide polymorphisms (SNPs), uninterpreted data that results from the analysis of the biological sample, and any information extrapolated, derived, or inferred therefrom” [72]. Such updated language can also inform which of these elements could be controlled, utilized for the public good, or patented. Although privacy may be hard to keep in a world of ubiquitous genetic, molecular, and data profiling, the statutes and laws can be updated as the methods and tools advance.

## Data Availability

Not applicable

## Abbreviations

GINA: Genetic Information Non-discrimination Act
GIPA: Genetic Information Privacy Act
ACA: Affordable Care Act
GDPR: General Data Protection Regulation
STRs: Short Tandem Repeats
CODIS: Combined DNA Index System
AIMs: Ancestry Informative Markers
HMP: Human Microbiome Project
PJC: Parliamentary Joint Committee
